# Methodology for Evaluating the Cutting Force of Planar Technical Blades Used in Flatfish Processing

**DOI:** 10.3390/mi12121516

**Published:** 2021-12-06

**Authors:** Bartosz Zieliński, Tomasz Chaciński, Danil Yurievich Pimenov, Krzysztof Nadolny

**Affiliations:** 1 Espersen Koszalin Sp. z.o.o., Mieszka I 29, 75-124 Koszalin, Poland; b.zielinski@espersen.com; 2 Department of Production Engineering, Faculty of Mechanical Engineering, Koszalin University of Technology, Racławicka 15-17, 75-620 Koszalin, Poland; tomasz.chacinski@tu.koszalin.pl; 3 Department of Automated Mechanical Engineering, South Ural State University, Lenin Prosp. 76, 454080 Chelyabinsk, Russia; danil_u@rambler.ru

**Keywords:** cutting force measurements, cutting blade, tool sharpness, food processing

## Abstract

In the food industry, there are many varieties of technical blades with different contours as well as different cutting edge geometries. The evaluation of the ability of technical blades to separate (cut) animal tissues is not a simple task and is usually based on the evaluation of the cutting effects in a technological process. This paper presents a methodology for evaluating the cutting force of technical blades used in food processing. A specially made test stand with numerical control was used in the study. Its application enabled a comparison of cutting force values for four different cutting edge geometries of planar knives used in the skinning operation of flat fishes. A unique feature of the conducted research was the use of a relatively high cutting speed value of *v_f_* = 214 mm/s, which corresponded to the real conditions of this process carried out in the industry. Obtained test results allow unambiguously choosing the most advantageous variant of knife geometry from among four different variants used for the tests. The results showed a clear relationship between the cutting force value and the value of the tip angle of the blades tested: for blades with the lowest tip angle, the lowest cutting force values were obtained.

## 1. Introduction

In the food industry, cutting organic materials is one of the stages in the production process to which great attention is paid. The fish industry is one of the industries where the cutting process with the use of technical blades is very much used. All kinds of production processes leading to a finished product are performed with the use of cutting tools [1,2]. Examples of operations include: the removal of undesirable elements of fish parts (tail, head, backbone, skin), and also giving the appropriate shape (cutting out specific portions) [2].

**Figure 1:**
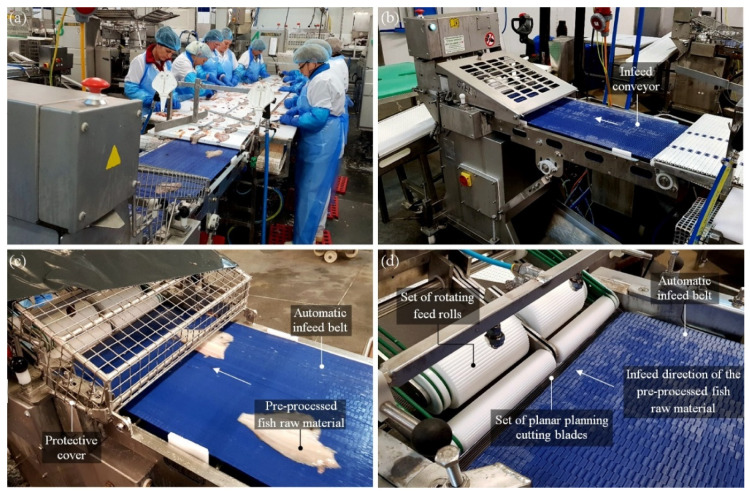
Overview of the skinning process of flat fishes from the *Pleuronectidae* family: (**a**) general view of technological line; (**b**) general view of automatic skinning machine ST600 (Steen F.P.M. International, Kalmthout, Belgium); (**c**) conveyor belt with pre-processed raw fish material; (**d**) mounting position of planar industrial cutting blades.

This study concerns the skinning operation, which is part of the fish fillet production process. It is an operation that involves separating the flesh of the fish and the skin from each other. An example of such an operation is the skinning of flat fish of the flounder family (*Pleuronectidae*) of Baltic origin and plaice (*Platessa Platesa Baltica*). The main role in the skinning operation is played by the technical blade, which separates the two organic materials. This tool significantly influences product quality and the continuity of the production process. The blade is installed in a skinning machine whose principle of operation is based on the mutual cooperation of the main transport roller feeding the raw material with the stationary knife separating the skin. Figure 1 shows the skinning process of flat fishes from the *Pleuronectidae* family as well as the automatic skinning machine ST600 (Steen F.P.M. International, Kalmthout, Belgium) [3,4,5].

Manufacturing plants strive to optimize the production process, which is understood as the desire to increase production efficiency and reduce costs while maintaining the desired product quality. From the point of view of durability of technical blades, the following problems are connected:

- Interruptions in the continuity of the production process: need for frequent replacement of worn-out blades, need for process monitoring, destruction of raw material;
- Financial aspect: one-time use of the tool, relatively large number of knife types, limited lifetime of knives.

Analyzing one of the most important characteristics of knives, which is their durability, we cannot forget about all kinds of disturbances that a blade encounters during its work. The irregularity of shape of the raw material itself (fillet), inclusions in the form of sand or scales, which are delivered from previous stages of production, have a very negative impact on the blade life. A humid and corrosive working environment and the presence of nitric and phosphoric acid are also detrimental. Premature damages (losses) of the cutting edge occur, which leads to interruptions in the production process realized in a stream system (production line). Therefore, it is necessary to conduct research in the field of possibilities of increasing the service life of technical blades, their modification and regeneration. However, the mentioned research areas require a precise assessment of the blade condition (mainly its sharpness) in order to verify the obtained results of the development works. Such verification can be carried out in the conditions of the real production process, but such a strategy is extremely expensive and introduces additional disturbances in plant operation. For research purposes, it is more advantageous to develop a methodology for evaluating blade sharpness in laboratory conditions as close as possible to the real conditions.

The laboratory evaluation of the sharpness of a technical blade intended for use in the food industry is not an easy task and is usually carried out using specialized cutting force measuring stands. The problem is not only a precise measurement of the cutting force, which can be treated as an indicator of the blade’s decohesion ability for the material being cut—and as a result its sharpness. An additional difficulty is the proper selection of the cutting material, which should as faithfully as possible correspond to the characteristics of biological tissues cut in food processing. Such tissues are very diverse and are characterized by a heterogeneous internal structure. In addition, the variables related to the shape and state (wear) of the blade and the conditions of its movement (kinematics and dynamics) must also be taken into account. All this makes the problem of determining the sharpness of technical blades still one of the important challenges in research on the development of food processing processes.

The literature on the subject includes papers presenting both analytical and computational results [6,7] as well as experimental studies in this field [8,9,10,11,12,13,14]. However, it seems that experimental research studies realized much higher accuracy at the current stage of development of computational methods with the use of special test stands.

The literature sources provide a collection of examples of measuring systems built especially for the purpose of assessing the sharpness of technical blades in the process of cutting of soft solids. McCarthy et al. in their works [7,8] proposed using a 50 kN Tinius-Olsen universal testing machine. In the lower part of the experimental position, the substrate material was mounted in anti-buckle clamps. The upper part of the rig held the blade via a blade handle, and the whole system was attached to the moving cross-head of the testing machine. The substrate was mounted flat and orthogonal with respect to the blade. As a substrate, a 2.25 mm thick polyurethane sheet with a Shore hardness of 40 A was used. This material was chosen as it has a similar constitutive form to that of most soft biomaterials (i.e., a J-shaped stress–strain curve), thus rendering this analysis suitable for applications in cutting biological tissues.

McGorry et al. proposed a different approach to the problem of testing blade sharpness [9,11]. They specified that the test stand must meet the following criteria:

- Should be portable and usable at the meatpacking plant;
- Should be simple and require no special skills to operate and allow evaluation of the entire cutting surface of the blade;
- The testing motion that should be representative of motions used in meat cutting (in most deboning operations, the cutter penetrates the meat with the tip of the knife and draws the blade through the meat).

As a result, a stand has been created that meets the above criteria, in which the knife to be tested was placed in a mounting fixture that rides on a pair of rails inclined at a 45° angle. The test knife was positioned in the fixture so that the long axis of the knife blade was approximately horizontal to the ground. A linear actuator drives the knife blade down the rail at a rate of approximately 40 mm/s. In this case, the material to be cut was polypropylene-coated fiberglass screening with a rectangular mesh (1.0 mm × 1.7 mm) and a strand thickness of 0.8 mm to obtain conditions similar to the cutting process of meat.

Diéguez et al. in their work [12], on the other hand, described a position built horizontally. The blade-holder was fixed in the *XY* plane permitting some regulation in the *Z* axis to accommodate in the test bank to different geometries of the blade. The sample goes to the blade with a constant and known velocity. This movement forward was produced by a linear actuator (a ball screw driven by a servomotor). Placed in blade-holder are two load cells, to measure the cutting force on the blade, both in the direction of the cut as well as the vertical direction. A similar arrangement was used by Brown et al. [13] in their study on cutting forces in foods and also by Marsot et al. [15] in their study on the sharpness of meat cutting blades.

Another example of a purpose-built test stand is the system described in the work of Gilchrist et al. [14]. The authors used cruciform test specimens, which were conveniently obtained by using a cruciform cutting die of arm width of 10 mm and arm length of 120 mm (ODC Tooling & Molds, Waterloo, ON, Canada). The specimen was held by two opposing pairs of clamps in a varyingly controlled manner to reproduce the range of unequal biaxial stresses that exist in vivo within skin. The blade of the knives was held in a separate clamp, which was in turn attached to a load cell of a uniaxial testing machine (Tinius Olsen Testing Machine Company, Redhill, UK). This load cell and the knife blade were aligned perpendicularly to the plane of the clamped skin specimens, and the testing machine was operated in a displacement mode of control. This meant that as the machine actuator forced the blade to indent and then penetrate a skin-stimulant specimen, the actuator velocity corresponded directly to the out of plane (i.e., downwards) displacement of the specimen. In addition, in this study, polyurethane of Shore hardness 40 A was used as a specimen material. It has a J-shaped stress–strain curve with a strength of approximately 17 MPa and a final stiffness of 22 MPa: this is qualitatively similar to biological skin. Moreover, it is isotropic, as attested by the superposition of the longitudinal and transverse test curves.

Blade sharpness is strongly related to the cutting force values obtained during the cutting process. However, one of the most important factors influencing cutting force is the geometry of the blade: mainly its wedge angle and tip radius. The literature sources have information referring to the influence of these parameters on cutting forces [7,9,16,17,18,19]. The results of most of the cited works show that an increase in wedge angle causes an increase in cutting force [17]. In addition, the value of the blade roundness radius has a similar effect on the cutting force, which was confirmed by works [16,18,19].

The described known systems for measuring the cutting force (which allows inferring blade sharpness) are characterized by the possibility of realizing cutting tests in a limited range of speeds. The authors have usually used relatively small values of cutting speed to obtain quasi-steady conditions. The present work deals with the process of skinning flat fish, which is realized in industrial conditions at speeds of the order of 100–300 mm/s. Therefore, the authors of this work decided to develop the original design of a test stand, which would enable evaluating the sharpness of different types of technical blades used in the process of skinning fish, while maintaining conditions as close as possible to the real process.

## 2. Materials and Methods

The aim of this study was to analyze the effect of differences in the cross-sectional outline of industrial cutting blades made of X39Cr13 steel used in the process of skinning flatfish of the *Pleuronectidae* family on cutting force. A series of tests were performed on a special test stand using blades with four different blade transverse outline geometries to determine the differences in cutting force between blade types.

### 2.1. Test Stand

**Figure 2:**
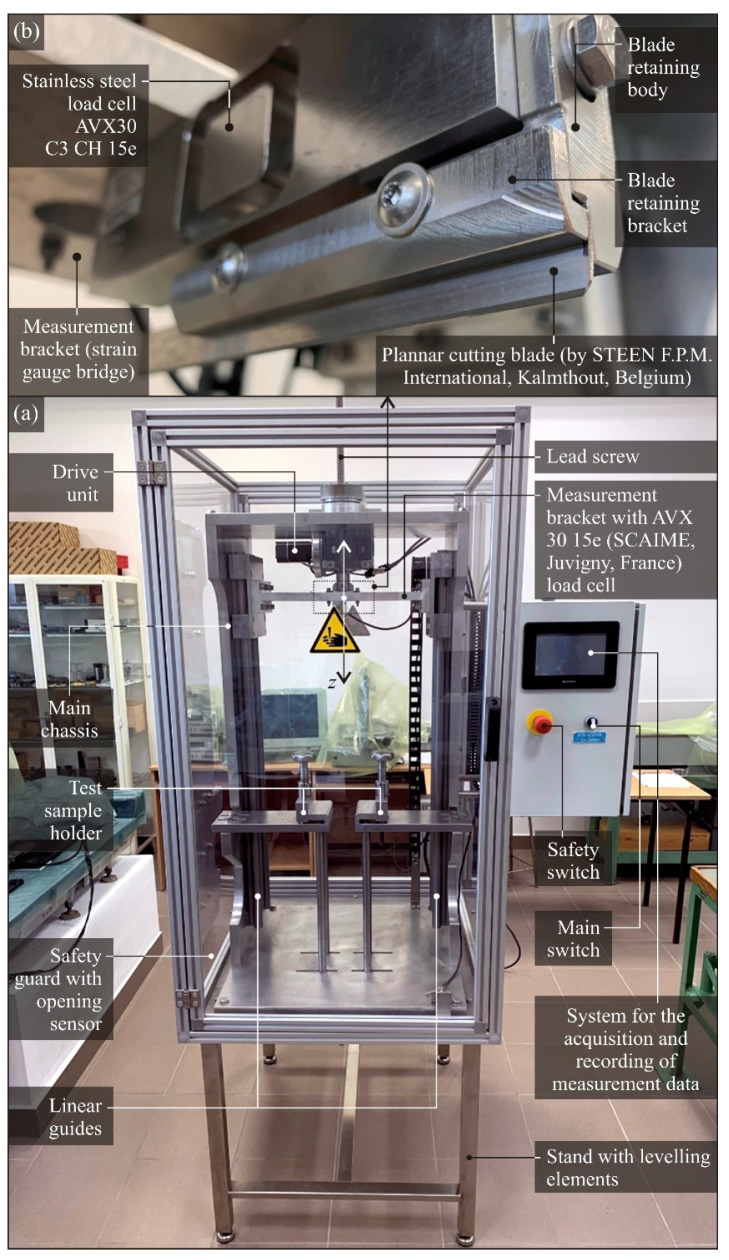
General view of the cutting force evaluation test stand with the most important functional modules indicated (**a**) and a view of fixing method of the blade to be assessed (**b**).

The original test stand is equipped with a fixture for clamping the test samples and cutting blades, as well as an operator interface from which the specified cutting speed *v_f_* can be set. Thus, at the developed test stand, it is possible to study the influence of the following cutting process variables: cutting speed *v_f_*, type and geometry of the blade, as well as the type and shape of the material being cut. Figure 2 shows a general view of the cutting force evaluation test stand with the most important functional modules indicated (Figure 2a) as well as a view of the fixing method of the blade to be assessed (Figure 2b).

The work of the measuring stand consists in putting in uniform feed motion the measurement bracket with the attached blade. The drive of measurement bracket is realized by the rotary motion of the motor and gearbox, which through a nut and a drive screw is converted into linear motion in the *Z* axis called the feed motion *f*. The cutting speed *v_f_* of the measurement bracket can be set in a very wide range of values (1–400 mm/s), while the positioning (return) motion is defined and cannot be changed.

The base of the cutting force measuring stand is a stable frame made of stainless steel profiles with Section 40 × 40 mm and wall thickness of 2 mm. It is supported on four M12 anti-vibration bases with the possibility of regulation. The table top made of stainless steel sheet with a thickness of 10 mm is an assembly element for the remaining modules of the stand.

The main body of the test stand (to which the actuators are attached) is made of a 20 mm thick aluminum sheet. The individual elements are fastened with M10 screws to form a rigid structure for the linear guides, drive system, and test samples holders.

The linear guides are the elements along which the main working movement is carried out. The guideways are attached to the main body by means of screws, along which the guide carriages of the measurement bracket with the load cell and the tested blade move.

The measurement bracket is a movable element that plays a very important role in the entire measuring stand. Guiding carriages are bolted to it, enabling rectilinear movement, and a drive system is attached to it. A properly configured drive system sets the measurement bracket in motion, on which the load cell with the holder of the tested blades is attached.

For correct measurement results, it is important to properly fix the samples to be cut. The sample holder is made in the form of clamping jaws, which are appropriately supported for the high rigidity of the system. The working elements (jaws) are properly profiled and made of PA6 aluminum sheet, which are clamped with M12 screws with ergonomic knobs for ease of use.

The measuring stand is equipped with an original control system made of components available on the market, which were properly selected and configured. The main element enabling the measurements is the AVX 30 15e load cell (SCAIME–Weighing & Measurement for Industry, Juvigny, France) with a working load range of 0–15 kg. Measurement data from the load cell are sent to the eNod4-T-DI00-000-SC signal amplifier from the same manufacturer. The processed signal is sent to the controller, where it is recorded using proprietary software. The operator panel is based on the USL-050-B05 programmable operator panel (Unitronics, Airport City, Israel) where the operating parameters are set and measurement results are collected. After the measurement, the recorded data can be digitally downloaded through the USB port for further analysis.

In order to maintain operator safety, the stand has been enclosed in a shield protecting access to moving parts during work. The shield has a door, which provides access to the mounting of tested blades and to the material being cut. The shield is made of 30 mm aluminum sections with 4 mm thick transparent polycarbonate plates sandwiched between them for good observation of the cutting test.

Table 1 shows the main technical parameters of the cutting force measurement station.

### 2.2. Tools and Workpieces

**Figure 3:**
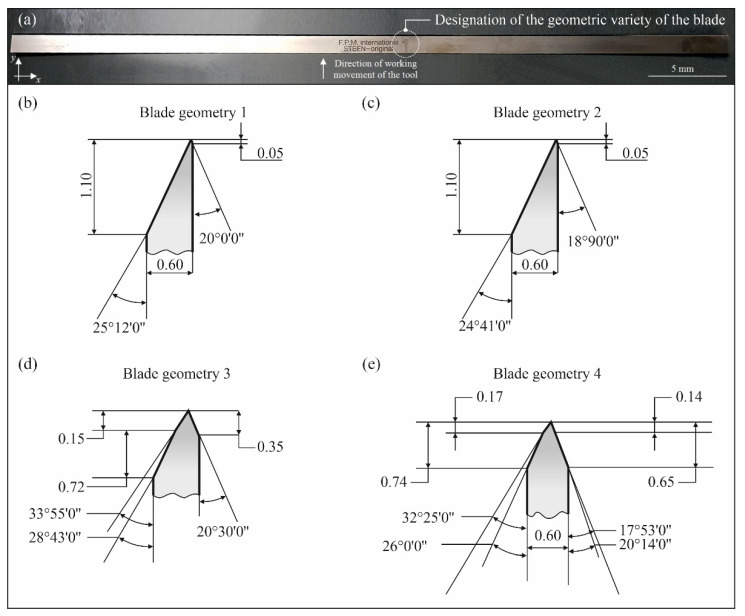
Overall view of the planar cutting blade used in experiments (**a**) and outline drawings of the cross-sectional geometry of the blades tested: (**b**) blade geometry 1; (**c**) blade geometry 2; (**d**) blade geometry 3; (**e**) blade geometry 4.

Four types of planar cutting blades dedicated for the flat fish skinning process (flounder *Platichthys flesus trachurus* and European plaice *Pleuronectes platessa*) differing in the axial outline of the blade (Figure 3) were used in the experiments. The blades were made of X39Cr13 high-carbon martensitic stainless steel (Kuno Wasser GmbH, Solingen, Germany for Steen F.P.M. International, Kalmthout, Belgium) and had the following overall dimensions: 459.5 mm × 12.3 mm × 0.6 mm. The blades were new (not used before) in the condition provided by the manufacturer. The physical properties of X39Cr13 steel was given in Table 2.

In most scientific works concerning the analysis of the basis of the cutting process of animal tissues, experimental research is carried out using materials imitating the properties of such tissues. Tests are usually not conducted with animal tissues due to difficulties in adapting the test stand and laboratory to maintain appropriate hygienic conditions for working with such material (sensitivity of measuring systems to water, detergents, and other cleaning agents required), difficulty in preparing homogeneous test samples, the need for proper storage of samples to prevent spoilage, difficult analysis of the surface of cut samples, etc. In the described studies, polyurethane (EKO INDUSTRIE Sp.z o.o., Słupsk, Poland) was selected as the cut material to test the cutting forces occurring in the process. This material was selected on the basis of research by other authors on the analogous cutting processes [7,8,14] described in Section 1 of this text. Table 3 lists the most important mechanical parameters of the polyurethane used in the shear force tests [20].

**Figure 4:**
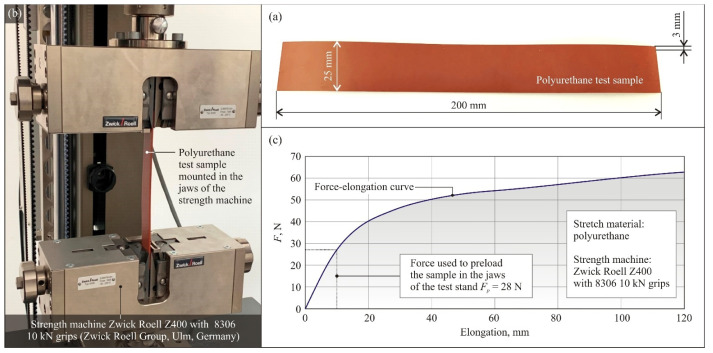
General view of test sample made of polyurethane (**a**), the strength characteristics of which (**c**) were determined by axial tensile test on a Zwick Roell strength machine Z400 8306 with 10 kN grips (Zwick Roell Group, Ulm, Germany) (**b**).

Test samples with dimensions of 200 mm × 25 mm cut from a 3 mm thick polyurethane sheet were prepared for testing. Figure 4a shows a general view of the test sample. In order to determine the force necessary to pre-tension the test sample in the fixtures, the characteristics of the test sample (Figure 4c) were determined in an axial tensile test on a strength machine Zwick Roell Z400 with 8306 10 kN grips (Zwick Roell Group, Ulm, Germany); see Figure 4b. The results of the measurements showed that in order to obtain the assumed pre-tension of the test sample resulting in its elongation by 10 mm, it is necessary to apply a force *F_p_* = 28 N to one of the test sample ends (Figure 4c). In this area, the force–elongation curve is linear. The *F_p_* = 28 N was applied each time to the test samples during clamping in the jaws.

### 2.3. Experiments Methodology

**Figure 5:**
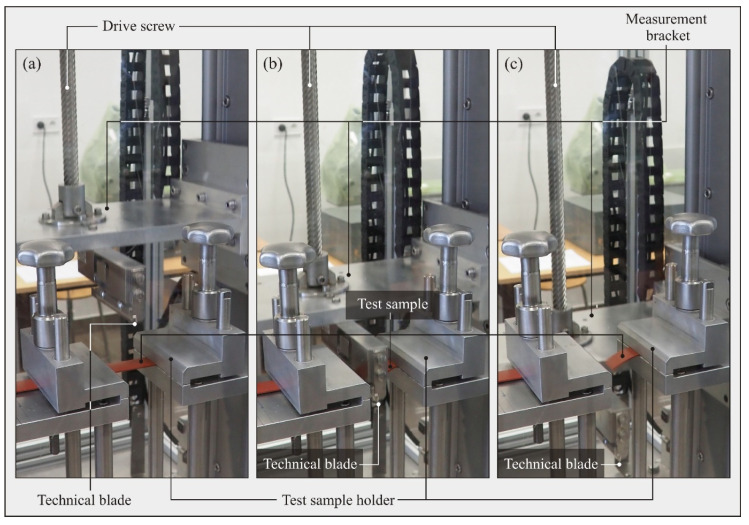
Visualization of the process of cutting through the test sample made of polyurethane: (**a**) initial stage—the blade approaches the test sample with a uniform motion at a remote speed; (**b**) second stage—cutting through the test sample; (**c**) third stage—completion of the test after moving the knife below the cutting zone.

The measurement consisted of clamping the specimen in a test sample holder, clamping the tested blade, and determining the speed of movement of the blade vice, which determines the speed of cutting the test sample. During the cutting process, the strain load cell, on which the holder and the blade are mounted, continuously measured the cutting force *F* transmitting the measured data during the blade movement from start to stop. The measurement data were transferred to a digital amplifier and then to the operator panel, where it was archived. Then, the recorded force distribution during the test could be digitally transferred to any software for further analysis. Figure 5 shows photographs taken during the three most important stages of the cutting test: initial stage—the blade approaches the test sample with uniform speed (Figure 5a); second stage—the sample is cut (Figure 5b); third stage—the test is finished after the blade is moved below the cutting zone (Figure 5c).

In order to achieve consistent positioning of each test sample to be measured, the left edge of the sample was aligned with the prepared distance marker, and the right jaw of the grip was clamped. Then, the sample was preloaded by applying a 28 N force to the left end of the sample (resulting in a 10 mm elongation), and the left grip jaw was clamped.

The research assumed a cutting blade feed rate of *v_f_* = 214 mm/s equal to that used in the production line of an industrial plant processing flat fishes (in this case, the speed was taken from medium-sized fish processing plant Espersen Koszalin Sp. z o.o., Koszalin, Poland).

The methodology for measuring the cutting force was carried out according to the following algorithm:

- Blade fixing;
- Fixing the test sample;
- Closing the measurement chamber door;
- Starting the main servomotor;
- Zeroing of the *Z*-axis by moving it to the upper extreme position;
- Entering the feed rate value *v_f_* = 214 mm/s;
- Release of the safety switch;
- Zeroing of the force sensor *F* reading with the *Tare* and *Max* = 0 functions;
- Start of the measuring procedure with the function *Start Velocity*;
- After the measurement has been completed, the safety stop was activated;
- Opening the chamber door and unclamping the cut sample by releasing the clamping jaws.

Then, the measurement data taken from the control module were converted using UniStream Data Converters Suite 1.0.17 software (Unitronics, Airport City, Israel). The converted files in *.xlsx format were further processed and analyzed using Excel spreadsheet version 2109 compilation 16.0.14430.20292 (Microsoft, Redmond, WA, USA).

For each of the 4 analyzed geometrical variants of technical blades, 3 cutting tests (3 repetitions) were carried out. Therefore, a total of 12 experiments were carried out in this study.

## 3. Results and Discussion

**Figure 6:**
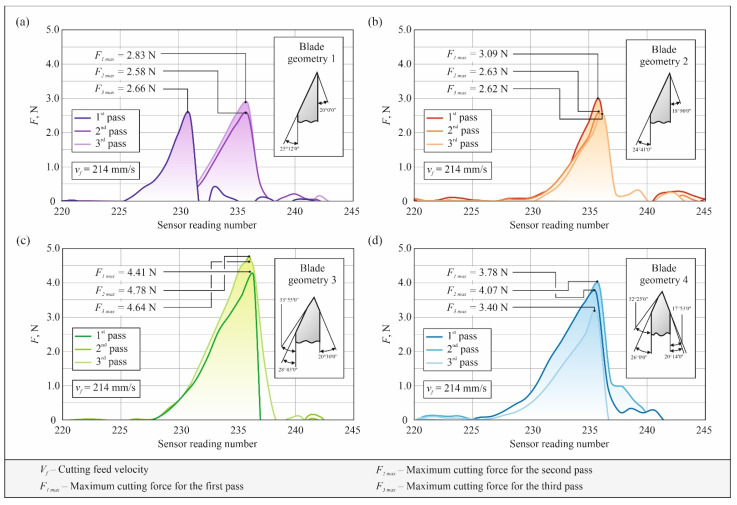
Collection of experimental results of cutting force *F* of knives with four different transverse blade outlines: (**a**) results of three repetitions for the knife with geometry 1; (**b**) results of three repetitions for the knife with geometry 2; (**c**) results of three repetitions for the knife with geometry 3; (**d**) results of three repetitions for the knife with geometry 4.

Figure 6 shows the results of the performed experimental tests of the cutting force *F* of knives with four different blade cross-sections geometry: for blade 1 (Figure 6a), 2 (Figure 6b), 3 (Figure 6c), and 4 (Figure 6d), respectively.

**Figure 7:**
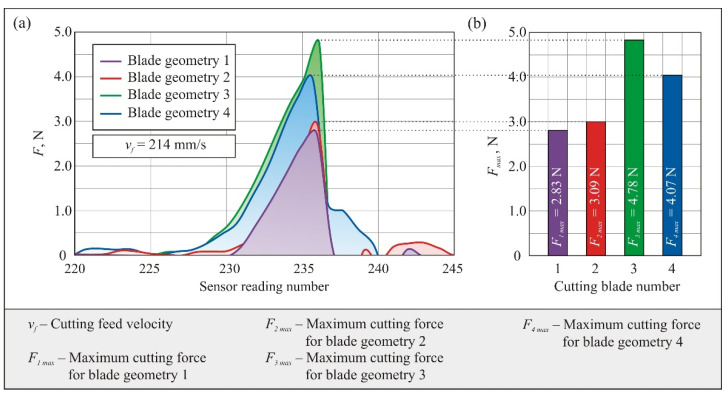
Summary of recorded plots of cutting force F for the four blade geometries evaluated—tests with maximum *F* values for each blade (**a**) and plot of maximum force values *F_max_* (**b**).

Additionally, in order to determine the effect of differences in blade geometry on the cutting force *F* values, the courses of changes in the measured value corresponding to the trials in which maximum values of *F* were obtained for each of the considered blade types are presented in a single graph (Figure 7a). Then, the maximum values *F_max_* for each of the blade geometries are presented in a bar chart (Figure 7b).

The obtained measurement results show significant differences in the cutting force *F* values between blades with geometries 1 and 2 and the other tools. The maximum force for blade 1 was *F_1 max_* = 2.83 N and for blade 2 was *F_2 max_* = 3.09 N; i.e., they differed only slightly (by about 9%). However, for the blade with geometry 4, the cutting force was 43% higher in relation to *F_1 max_* (*F_4 max_* = 4.07 N). The highest cutting force was recorded for geometry 3 (*F_3 max_* = 4.78 N), and it was about 69% higher than *F_1 max_* (Figure 7b).

When analyzing the values of the maximum cutting force for the studied geometric varieties of blades, a clear relationship can be seen with the value of their tip angle. Knives of types 1 and 2 are characterized by the lowest value of the tip angle amounting from 43°31’ (knife with geometry 2) to 45°12’ (knife with geometry 1). For the knife type 4, the value of this angle is already 52°25’, and for the knife with geometry 3, it is the highest among the selected tools: 54°25’. Therefore, it can be concluded that a decrease in the value of the tip angle favors a reduction of the force in the cutting process. At the same time, such an action will reduce the strength of the blade and increase its susceptibility to deformation and chipping in case of contact with hard inclusions or debris in the material being cut. This means that an optimum compromise must be sought to ensure both the required mechanical strength and relatively low cutting forces conducive to long-term stable operation of the tool. The influence of the blade radius value was neglected in the considerations. It was assumed that in case of all assessed knife geometries, the radius of rounding of the blade is similar due to the fact that tools of the same type (planar knives with dimensions 459.5 mm × 12.3 mm × 0.6 mm), made of the same material (X39Cr13 steel) and by the same manufacturer (Steen F.P.M. International, Kalmthout, Belgium) were selected for evaluation.

The differences in cutting force values indicate that the different shape of the geometry of the cross-sectional contour of the blade significantly affects the cutting force, which may result in different tool life in actual production processes. It seems that the aim should be to minimize the value of the cutting force, which is the resistance of the cutting material subjected to decohesion through the mechanical force exerted on the cutting blade. In this context, the results obtained with blades of geometries 1 and 2 should be considered as the most favorable. From the analysis of their cross-sectional outline shown in Figure 3, it can be seen that they differ very little from each other, which confirms the similar values of the recorded cutting forces in the experimental tests carried out.

However, it should be remembered that the evaluation of the cutting ability (sharpness) of a blade expressed by the value of the cutting force is not the only criterion for assessing the operational effectiveness of a tool of this type. For this reason, laboratory tests should be supplemented in the future with operational tests in conditions of the real process of processing raw fish in order to determine the phenomena determining the blade wear and to determine the influence of the blade geometry on its service life.

## 4. Conclusions

On the basis of the construction, execution, and research work carried out, the following specific conclusions were formulated for the developed methodology for evaluating the cutting force of planar technical blades used in flatfish processing.

- In order to reproduce as accurately as possible the industrial conditions of the cutting process carried out in the processing of flat fish, it was necessary to design and develop a special test stand characterized by a very large variation range of the cutting speed *v_f_* = 1–400 mm/s.
- The test stand was characterized by numerical control and digital recording of measurement data with high resolution and frequency, ensuring full repeatability of the tests.
- A unique feature of the conducted research was the use of a relatively high cutting speed value of *v_f_* = 214 mm/s, not encountered before in the literature, which corresponded to the real conditions of this process carried out in a medium-sized fish processing plant (Espersen Koszalin Sp. z o.o., Koszalin, Poland).
- The results of the tests allowed selecting the blade geometries 1 and 2 as the most advantageous ones, providing the lowest values of cutting force *F* among the set of blades used for the tests.
- The results showed a clear relationship between the cutting force value and the value of the tip angle of the blades tested: for blades with the lowest tip angle value (geometry 1: 45°12′, geometry 2: 43°31′), the lowest cutting force values were obtained (geometry 1: *F_1 max_* = 2.83 N, geometry 2: *F_2 max_* = 3.09 N). Increasing the tip angle by approximately 20% to 52°25’ (geometry 4) resulted in a 43% increase in the cutting force with respect to *F_1 max_* (F4 max = 4.07 N). The highest cutting force value was recorded for the knife with geometry 3 (*F_3 max_* = 4.78 N, which is about 69% more than *F_1 max_*), which also had the largest tip angle of 54°25’ (approximately 25% more than for geometries 1 and 2).
- A decrease in the value of the tip angle favors a reduction of the force in the cutting process; however, lowering the tip angle reduces the strength of the blade and increases its susceptibility to deformation and chipping in case of contact with hard inclusions or debris in the material being cut. To ensure both the required mechanical strength and relatively low cutting forces, an optimum compromise must be reached to long-term stable operation of the tool.
- The tests described are of a laboratory nature, and it is not possible to reproduce under such conditions the influence of disturbing factors occurring randomly and with varying intensity in a real production process. Therefore, test results obtained on the test bench described above must ultimately be verified by trials conducted under industrial conditions on a flat fish skinning production line.
- Further research is planned to determine the effect of blade wear and blade regeneration process on cutting forces. The test stand developed will also allow future research to be conducted into the effect of cutting speed *v_f_* and the type and thickness of the material being cut on the cutting force.

## Figures and Tables

**Table 1:** Technical parameters of the cutting force measurement station.

Parameter	Value
Power supply	AC 230 V/50 Hz
Safety systems	Sensor for measuring chamber door closing, emergency stop
Control panel	Touch control panel USL-050-B05 (Unitronics, Airport City, Israel)
Load cell sampling rate	Interval 3–4 ms (250–333 Hz)
Working load range of the load cell	0–15 kg
Feed rate during measurement	1–400 mm/s
Cutting force measuring unit	Grain
Communication interface, transfer of measurement results	USB-A, via digital flash drive

**Table 2:** The physical properties of X39Cr13 high-carbon martensitic stainless steel.

Country	EU	USA	Germany	France	England	Italy	China	Poland
Standard	EN	–	DIN, WNr	AFNOR	BS	UNI	GB	PN
Designation	X39Cr13	420	X39Cr13	Z40C13	X39Cr13	X40Cr14	4C13	4H13
Physical properties								
Thermal expansion		Modulus of elasticity	Poisson number	Electrical resistivity	Electrical conductivity	Specific heat	Density	Thermal conductivity
10^−6^∙K^−1^		GPa	v	Ω∙mm^2^/m	S∙m/mm^2^	J/(Kg∙K)	Kg/dm^3^	W/(m∙k)
10.5		215	0.27–0.30	0.55	1.82	460	7.70	30

**Table 3:** Key mechanical properties of the polyurethane used in the cutting force tests [20].

Parameter	Value
Tensile strength DIN 53504	45–50 MPa
Elongation at break DIN 53504	450–680%
Abrasion DIN 53516	25–50 mm^3^
Elastomer density	1.25–1.30 g/cm^3^
Shore hardness DIN 53505	55–95° ShA
Maximum temperature resistance	80 °C
